# Risk for Travel-associated Legionnaires’ Disease, Europe, 2009

**DOI:** 10.3201/eid1811.120496

**Published:** 2012-11

**Authors:** Julien Beauté, Phillip Zucs, Birgitta de Jong

**Affiliations:** European Centre for Disease Prevention and Control, Stockholm, Sweden; European Legionnaires’ Disease Surveillance Network, Stockholm

**Keywords:** Legionnaires' disease, Legionella, legionellosis, pneumonia, airborne, respiratory, waterborne, travel, risk, surveillance, Europe, bacteria

## Abstract

Legionnaires’ disease is underreported in Europe; notification rates differ substantially among countries. Approximately 20% of reported cases are travel-associated. To assess the risk for travel-associated Legionnaires’ disease (TALD) associated with travel patterns in European countries, we retrieved TALD surveillance data for 2009 from the European Surveillance System, and tourism denominator data from the Statistical Office of the European Union. Risk (number cases reported/number nights spent) was calculated by travel country. In 2009, the network reported 607 cases among European travelers, possibly associated with 825 accommodation sites in European Union countries. The overall risk associated with travel abroad was 0.3 cases/million nights. We observed an increasing trend in risk from northwestern to southeastern Europe; Greece had the highest risk (1.7). Our findings underscore the need for countries with high TALD risks to improve prevention and control of legionellosis; and for countries with high TALD risks, but low notification rates of Legionnaires’ disease to improve diagnostics and reporting.

## Abstract 2

Infections increased in a southeastern direction, with highest risk in Greece.

Legionnaires’ disease (LD) is a severe multisystem disease, typically manifesting as pneumonia; it is caused by gram-negative bacteria, *Legionella* spp., which are found in freshwater environments worldwide, and can be can be transmitted by aspirating contaminated drinking water or by inhaling airborne droplets from contaminated sources such as building ventilation units and spas (*1*). A travel-associated Legionnaires’ disease (TALD) surveillance system at the European Union (EU) level has been in place since 1987 (*2*); EU surveillance of Legionnaires’ disease, regardless of patient travel history, began in 1996 (*3*). The rationale for the surveillance of travel-associated cases of Legionnaires’ disease (TALD) is that it enables the timely detection of clusters and the identification of the source of infection, which would otherwise probably remain undiscovered (*4*). Since April 2010, both surveillance systems have been operated by the European Legionnaires’ Disease Surveillance Network (ELDSNet) under the coordination of the European Centre for Disease Prevention and Control (ECDC).

Since 2005, 5,000–6,000 cases of Legionnaires’ disease have been reported each year by the 27 EU Member States, Iceland, and Norway (*5*,*6*). Annual case-fatality rates of notified Legionnaires’ disease have been ≈10%, and ≈20% of reported cases are travel-associated (*5*–*7*), which is similar to the situation described in the United States (*8*). There is evidence that the risk for Legionnaires’ disease might be higher under certain environmental conditions; warm and wet weather has been associated with higher incidence rates in the Netherlands and the United Kingdom (*9*,*10*). Using that evidence, Hicks et al. reported that an increased case count would be expected after heavy rains during the warm season (*11*). However, in Europe, an increasing trend from northern to southern countries is not clearly evident in surveillance data.

Because many cases of Legionnaires’ disease might go undiagnosed by clinicians, and because some clinicians do not report confirmed cases to national authorities, surveillance systems are likely to miss a sizeable proportion of Legionnaires’ disease deaths (*1*). The ECDC estimated that as few as 10% of Legionnaires’ disease cases are reported in Europe and reported that notification rates differ substantially across countries and that causes of undernotification are likely to vary (*6*). Because notification rates tend to reflect the quality of national surveillance rather than the local risk for Legionnaires’ disease, it is difficult to estimate and compare risk for Legionnaires’ disease across countries.

The surveillance of Legionnaires’ disease associated with international travel might, at least partly, overcome this limitation. A similar approach has been used in previous studies to estimate and compare the risks for foodborne and waterborne diseases in Europe (*12*,*13*). Tourism is a massive industry in Europe, and its summer peak coincides with the peak of Legionnaires’ disease. Cases of TALD in travelers who contract the disease in the country they visit are mostly reported by the patient’s country of residence independent of the quality of surveillance in the travel destination country. These cases may therefore enable not only a better estimation of local disease risk but also an assessment of the local quality of reporting. Finally, Legionnaires’ disease surveillance in Europe has traditionally focused more on TALD, because of the added value of improved prevention and control of international clusters. TALD surveillance in Europe can therefore be assumed to be less prone to underascertainment than the surveillance of Legionnaires’ disease not associated with travel.

This analysis is intended to assessing the risks for TALD in European countries on the basis of travel patterns. A secondary objective is to provide an estimate of the extent of underascertainment by country of destination.

## Methods

### Data and Definitions

In travel medicine, the use of surveillance data is a valid method for calculating absolute risk estimates in travelers (*14*). Notification of Legionnaires’ disease cases is mandatory in all 27 EU Member States (notification has been mandatory in the United Kingdom since 2010) and Iceland and Norway, the 2 European Economic Area (EEA) member states that form part of ELDSNet. TALD cases were reported during 2009 to ECDC by most ELDSNet members. Germany did not participate in TALD reporting during this period. Reporting was performed on a daily basis through the web-based European Surveillance System, known widely as TESSy. TALD cases must conform to the official EU case definition of Legionnaires’ disease (*15*), and case-patients must have a history of travel, i.e., at least 1 night spent in commercial accommodation within the EU/EEA countries, away from their residence (including travel within their home country) within the incubation period of Legionnaires’ disease (2–10 days before disease onset). Date of onset and date of travel are carefully checked.

For the purpose of this analysis, we included TALD cases with disease onset in 2009 and retrieved the patients’ places of residence and travel history from TESSy. Cases counted were restricted to European residents traveling in EU/EEA countries. The travel history could include more than 1 travel destination or accommodation site, each of which was considered a potential source of exposure. Therefore, a case would be counted in more than 1 country if the case-patient’s travel history involved accommodation sites in various countries. The exact duration of stay was not taken into account in the analysis. Tourism denominator data for 2009 were obtained from the Statistical Office of the European Union (Eurostat) (*16*). We used the number of nights spent in tourist accommodations by Europeans traveling in their home countries and in foreign countries within Europe. The number of nights spent was also categorized by a traveler’s country of residence and destination country.

### Analysis

Case-patients were described by age, sex, month of illness onset, travel destination, type of travel, and type of accommodation. To estimate the exposure rate, we used the number of nights spent by country of destination. For a given country of destination, risk for TALD was defined as the number of cases divided by the number of nights spent in that country by all travelers (population at risk). To allow comparisons of risk by country of origin, we excluded case-patients for which the reporting country was different from the country of residence. Risk at country level was calculated only for countries with at least 10 cases reported. To estimate underascertainment, a set of reference reporting countries with at least 20 cases reported in nondomestic travelers was chosen. We then compared the risk for Legionnaires’ disease of travelers originating from these reference countries to the risk for domestic travelers, calculating incidence ratios with 95% CIs for each destination country. Last, on the basis of the risk for travelers from the reference countries, we estimated the number of cases expected in each travel destination country. These estimates were obtained by multiplying the risk in reference countries with the total number of nights spent in tourist accommodations by all travelers in a given destination country.

## Results

### Cases and Sites

**Figure 1:**
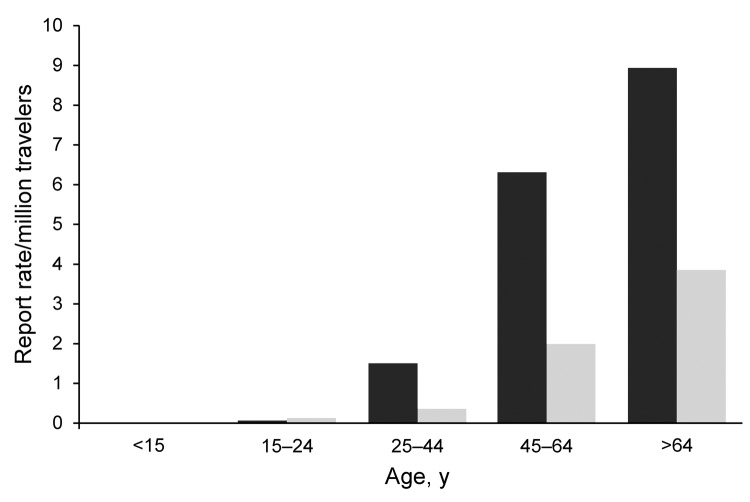
Notification rate of travel-associated Legionnaires’ disease (n = 607), by age group and sex, European Union/European Economic Area, 2009. Black bar, male case-patients; light gray bar, female case-patients.

In 2009, 607 TALD cases were reported among European residents traveling in EU/EEA countries, of which 363 (60%) were related to domestic travel (Table 1). Travel histories involved 825 accommodation sites in 24 countries. The average number of sites per case-patient was 1.4 (range 1–7); a similar distribution was calculated among countries. As travel destinations, France, Italy, and Spain accounted for 72% of cases and sites. Of the 825 sites involved, 447 (54%) had been occupied by domestic travelers. Notification rates increased with age and were higher in male case-patients for all age groups (Figure 1). The male:female ratio for case-patients was 2:7 and the median age at date of onset was 61 years (interquartile range 51–69 years). All case-patients were >15 years of age; 40% were >65 years of age. The seasonal distribution of cases peaked in September; the date of onset for 48% was during the third quarter of the year. Hotels were the most common accommodation sites associated with TALD, accounting for 578 (70%) of 825 sites of known type. This proportion applied to sites associated with domestic travel and sites associated with foreign travel. Other accommodation sites reported were campsites (8%), private accommodations rented for commercial purposes (6%), apartments (5%), cruise ships (<1%), and other accommodations (10%).

**Table 1:** Cases of travel-associated Legionnaires’ disease by country of residence and destination country, Europe, 2009*

Country of residence	Reported no. cases by destination country				
	Italy	France	Spain	Other countries	Total
Italy	140	5	2	3	149
France	14	106	5	10	133
United Kingdom	12	6	33	80	120
Netherlands	14	11	10	58	72
Spain	4	2	42	8	51
Denmark	9	1	1	15	22
Austria	7	1	0	7	14
Sweden	2	2	2	8	13
Other countries	7	3	3	21	33
Total	209	137	98	210	607

*A case-patient could have a travel history involving >1 country.

### Travel Patterns

In 2009, two billion nights were spent in tourist accommodations in EU/EEA countries by travelers who resided in Europe. Of those nights, 46% were spent in France, Italy, and Spain (Table 2). Overall, travel within the tourists’country of residence represented 66% of all nights spent in tourist accommodations. Among Europeans >65 years of age, the proportion of domestic travelers (19%) was higher than the proportion of travelers who visited other countries (15%; p<0.01). Conversely, the proportion of travelers in the 45–64 year age group was lower in domestic travelers (31%) than in those traveling to other countries (33%; p<0.01). Data at destination country level were not available.

**Table 2:** Cases of travel-associated Legionnaires’ disease and risk per million nights in European domestic and nondomestic travelers, Europe, 2009*

Destination	No. (% cases in domestic travelers)	No. nights spent by travelers, millions (% by domestic travelers)	TALD risk in cases/million nights	
			Domestic travelers	Nondomestic travelers
Italy	209 (67)	330 (64)	0.66	0.58
France	137 (77)	272 (72)	0.54	0.41
Spain	98 (43)	328 (45)	0.28	0.31
United Kingdom	45 (98)	228 (80)	0.24	0.02
Greece	34 (0)	58 (33)	0.00	0.88
Germany	28 (0)	296 (88)	0.00	0.79
Netherlands	21 (86)	79 (76)	0.30	0.16
Portugal	20 (20)	41 (47)	0.21	0.73
Austria	20 (15)	94 (33)	0.10	0.27
Other countries	42 (14)	314 (72)	0.03	0.33
Total	607 (60)	2,039 (66)	0.27	0.36

*A case-patient could have a travel history involving >1 country. TALD, travel-associated Legionnaires’ disease.

### Risk for TALD

Calculating on the basis of 2 billion nights spent in Europe by European residents and 607 cases, the average risk for TALD in Europe in 2009 was 0.30 cases/1 million nights (95% CI 0.27–0.32). The highest risk for domestic travelers was in Italy (0.66 cases/1 million nights), followed by France (0.54) and the Netherlands (0.30) (Table 2). On average, the risk for nondomestic travelers (0.36/1 million nights) was 1.3 × the risk for domestic travelers (0.27 cases/1 million nights). France, Italy, the Netherlands, and the United Kingdom were the only countries with higher risks for domestic travelers than for nondomestic travelers. The average risk in nondomestic travelers ranged from 0.02 cases/1 million nights in the United Kingdom to 0.88 cases/1 million nights in Greece.

### Underascertainment of Cases

**Figure 2:**
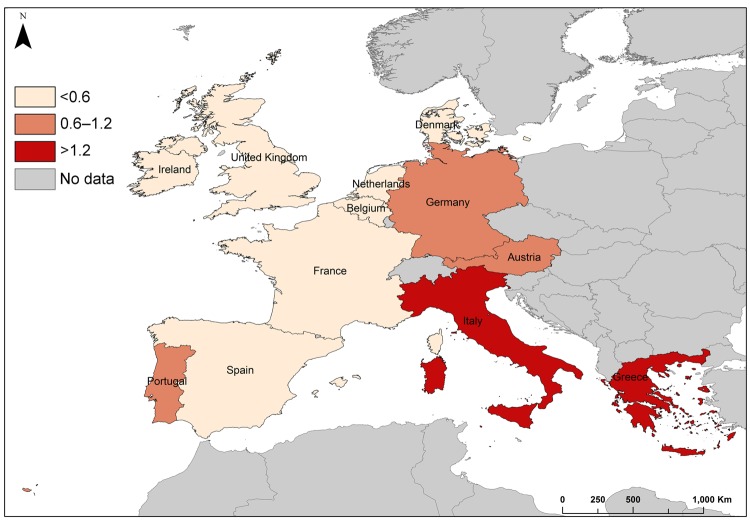
Risk for travel-associated Legionnaires’ disease in residents of Denmark, France, the Netherlands, and the United Kingdom who traveled to countries in Europe. Risk is displayed in countries where travelers spent at least 5 million nights in commercial accommodations during 2009, European Union/European Economic Area (in cases per million nights).

The United Kingdom, the Netherlands, France, and Denmark reported the highest numbers of cases in nondomestic travelers: 81, 72, 28, and 21 cases, respectively. These countries also reported the highest rates for nondomestic travelers, ranging from 0.62 to 1.24 cases/1 million nights. Sweden’s reported rate (0.74 cases/million nights) was within this higher range, but was based on 13 cases reported. The United Kingdom, the Netherlands, France, and Denmark were selected as reference reporting countries with a pooled overall risk of 0.55 cases/million nights and a pooled risk of 1.68 cases/1 million nights when traveling to Greece (Table 3). The risk for travelers from these reference countries becoming infected by *Legionella* was greater when traveling in Greece, Portugal, Germany, Italy, Austria, Spain, and France than when traveling in the United Kingdom (Table 3). Risk was high in southeastern countries (Greece and Italy), moderate in southwestern and central Europe (Austria, Germany, and Portugal), and low in northwestern countries (Figure 2). Based on these figures, 1,127 TALD cases would be expected to be reported in European residents traveling in Europe (Table 3). The highest levels of underascertainment were observed in Greece, Portugal, and Austria. Germany was not taken into account because cases in domestic travelers were not reported.

**Table 3:** Expected risk for Legionnaires’ disease in European travelers to nondomestic destinations in Europe, based on reference data reported by the United Kingdom, the Netherlands, France, and Denmark, Europe, 2009*†

Destination	Risk for travelers to destination (cases/million nights)	Incidence ratio (95% CI)	Total no. cases	
			Reported	Estimated
Greece	1.68	7.2 (4.2–12.2)	34	98
Italy	1.40	6.0 (3.9–9.2)	209	463
Germany	1.19	5.1 (2.9–8.7)	22	353
Portugal	1.06	4.6 (2.1–9.0)	20	44
Austria	1.01	4.4 (2.2–8.2)	20	95
Spain	0.57	2.5 (1.6–3.8)	98	188
France	0.53	2.3 (1.6–3.3)	137	145
Netherlands	0.33	1.4 (0.8–2.4)	21	26
United Kingdom	0.23	1.0 (Ref.)	45	53
Other countries	0.90	3.9 (2.3–6.4)	42	282
Total	0.55	NA	607	1,127

*A case-patient could have a travel history involving >1 country. Ref., referent; NA, not applicable. †In the United Kingdom, reporting of Legionnaires’ disease became mandatory in 2010.

## Discussion

We report risk estimates of TALD for domestic and international travelers in Europe. These results are valuable because sentinel studies that rely on travel medicine departments may not report on TALD (*17*,*18*). In addition, most studies focus on travelers who are returning from developing countries (*19*). Logarithmic scales of health risk among travelers, as provided by Steffen et al. (*20*), need regular updates. On the basis of the most recent data, collected during 2003 in Europe (*2*), the TALD risk was estimated to be between 0.0001% and 0.001% per month of stay in developing countries. The highest risk estimate that we found was 1.68 cases/1 million nights (Greece), which corresponds to an incidence rate of 0.005%/month, which is >5 × higher than the aforementioned estimate. Our risk estimates ranged from 0.001%/month in the United Kingdom to 0.005%/month in Greece and Italy.

It is highly probable that a similar risk would be found in other Mediterranean countries that share similar climate and tourist facilities. The seemingly higher risk among nondomestic travelers may reflect a better awareness of clinicians when investigating pneumonia in a patient who is returning from a trip abroad. Indeed, clinicians might be more likely to ask for additional tests, including urinary antigen, in patients with a recent history of travel abroad. It could also reflect, to some extent, better reporting in northern European countries, the residents of which tend to spend their vacations in southern Europe. Another hypothesis would be that domestic travelers have a better immunity against local strains. The low TALD risk in nondomestic travelers to the Netherlands and the United Kingdom could be explained by the fact that both countries receive proportionately far fewer and younger tourists than Mediterranean countries.

This study clearly indicates an increasing risk of TALD from northwestern to southeastern Europe. Greece did not report any domestic TALD cases in 2009, but the risk estimate for nondomestic travelers who had visited Greece was the highest. This discrepancy likely reflects inadequate disease diagnosis or reporting in Greece. When geographic latitude was considered, Germany posed a higher TALD risk than expected, and Spain appeared to have a lower risk than expected. The seemingly higher risk in Germany could be associated with travelers from the Netherlands, who accounted for nearly one third of all nights spent by nondomestic travelers to Germany, and whose cases were likely to be reported to their health authorities. Conversely, German travelers accounted for nearly one third of all nights spent by nondomestic travelers in Spain; because Germany did not participate in the European TALD surveillance, any cases that may have occurred in German travelers were not reported.

Nevertheless, this relatively high risk observed in nondomestic travelers who visited Germany might confirm a report of an increasing trend in France from west to east in 2010 (*21*). Heavy rains during the warm season in continental climates might have favored the growth of *Legionella* spp. The magnitude of TALD underascertainment in Austria, where German travelers accounted for 60% of all nights spent by nondomestic travelers, demonstrates the effect that the lack of participation in reporting by Germany had on epidemiologic studies of the EU/EEA (As of September 2012, Germany participates in the European TALD surveillance). The use of a pooled risk estimate as reference suggests that underascertainment of TALD remains substantial in Europe and concerns all nonreference countries. This conclusion is consistent with results of capture-recapture studies conducted in some European countries to assess the extent of undernotification (*22*–*24*). According to our study, ≈1,100 TALD cases would be expected annually in the EU/EEA.

This study has some limitations. First, it was assumed that travel habits were similar among all European travelers. Duration of stay could affect the likelihood of exposure while traveling and could vary across countries, age groups, and type of travel. Second, because of the lack of data on age group distribution among travelers, the calculation of risk could not be standardized by age. We know that domestic travelers are older and therefore more prone to be infected by *Legionella* spp. However, the proportions of Europeans >65 years of age remained comparable among domestic travelers and those traveling outside their home country. In addition, distribution of travelers by age groups may substantially differ across countries. Third, the study clearly highlights the lack of data for Eastern European countries, probably because tourism is lower than elsewhere in Europe and because TALD is under-identified. Increasing numbers of tourists and improving local clinical and diagnostic awareness might help estimate the TALD risk in this part of Europe in the future.

Further analyses should confirm these results over time. Larger datasets could allow adjustment for age, seasons, and accommodation types. In large countries, regional stratification would also be valuable since risk may differ within a country.

In conclusion, these European TALD risk estimates can provide data for several purposes. First, they may help raise clinicians’ awareness and enhance reporting in countries where risk for Legionnaires’ disease and TALD is high, but reporting rates are low. Second, they could serve as a basis for monitoring future trends. Considering global warming, increasing use of manmade water systems, and an aging European population, Legionnaires’ disease and TALD incidence might also be expected to rise.
